# Impact of zinc oxide on gut health, immunity, and growth in weaned piglets: exploring potential modes of action

**DOI:** 10.3389/fvets.2025.1645900

**Published:** 2025-09-01

**Authors:** Zacharia Waithaka Ng’ang’a, Nuria Tous, Maria Ballester, Jakob Leskovec, Beatriz Jimenez-Moya, Raúl Beltrán-Debón, David Torrallardona, Joan Tarradas

**Affiliations:** ^1^IRTA, Animal Nutrition, Mas Bové, Constantí, Catalonia, Spain; ^2^MoBioFood Research Group, Department of Biochemistry and Biotechnology, Universitat Rovira i Virgili, Tarragona, Spain; ^3^IRTA, Animal Breeding and Genetics, Torre Marimon, Caldes de Montbui, Catalonia, Spain

**Keywords:** zinc oxide, gut health, gene expression, immune response, inflammation

## Abstract

Zinc oxide (ZnO) has been used at pharmacological levels to promote gut health and growth performance in the critical postweaning (PW) phase of piglets. The pharmacological use of ZnO in piglet diets has been banned in Europe and other countries due to antimicrobial resistance and environmental concerns. Therefore, understanding its mode of action, including its molecular mechanisms, is crucial for developing effective and sustainable alternatives. We investigated the mechanisms by which dietary supplementation with 3,000 mg/kg ZnO supports gut health and improves growth performance of piglets during the first 14 days PW. During the 2 weeks of trial (0–14 d PW), ZnO fed piglets had higher average daily gain (165 vs. 123 g/d; *p* < 0.01), and tended to have increased average daily feed intake (204 vs. 181 g/d; *p* < 0.1) and improved gain-to-feed ratio (0.669 vs. 0.774; *p* < 0.05) compared to control piglets. Feces from piglets in the ZnO group were also more consistent during the 2 weeks of trial (*p* < 0.01). At day 14 PW, ZnO piglets had lower calprotectin concentrations in serum (*p* < 0.01). Dietary ZnO downregulated several genes, involved in immune, oxidative and inflammatory responses, in jejunal (*GPX2, REG3G, IL-8*, *IL-6*, *IL-22*, and *TGFβ1*) and ileal (*REG3G*, *IL-17A*, *IL-1β*, and *TLR2*) mucosa (*p* < 0.05). It also downregulated the expression of the zinc transporter *SLC39A4*, that is associated with zinc homeostasis, in both tissues. Notably, *PPARGC1A,* which promotes energy production and lipid metabolism through fatty acid oxidation, was upregulated by ZnO in ileum. In conclusion, the current results suggest that high dietary levels of ZnO reduce the expression of inflammatory cytokines, the oxidative enzyme *GPX2*, pathogen recognition proteins, and zinc transporters while promoting the expression of *PPARGC1A* gene related with energy metabolism in the intestine. Therefore, ZnO can facilitate a smoother weaning transition to reduce weaning related gut health disturbances, ultimately contributing to gut homeostasis and improved performance.

## Graphical abstract

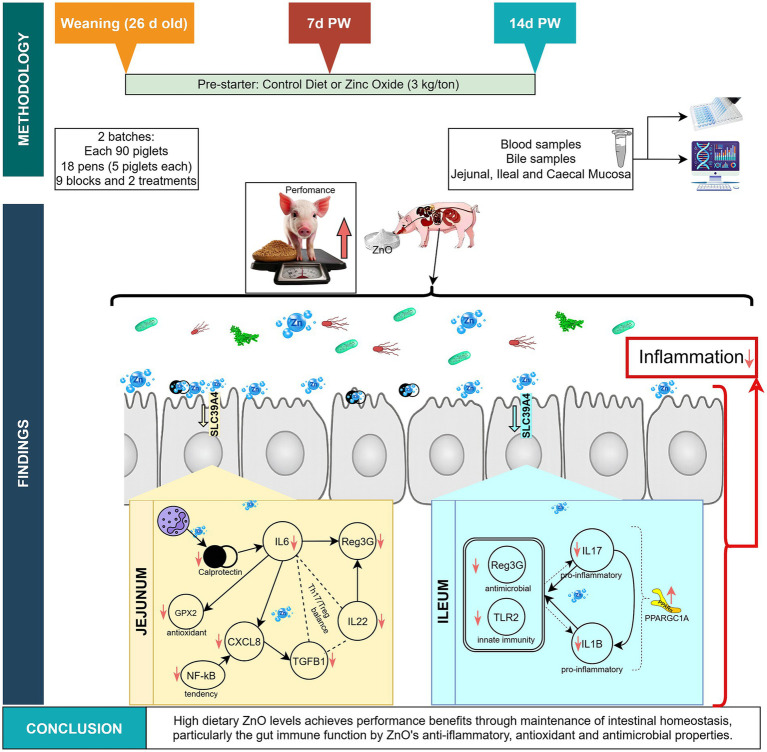

Zinc oxide (ZnO) supplementation (3 kg/ton for 2 weeks post-weaning) improved piglet performance and gut homeostasis by modulating immune and oxidative stress responses in both jejunum and ileum. ZnO reduced calprotectin concentration in blood, which is normally released by activated neutrophils to bind zinc and stimulate inflammation. In jejunum, ZnO appeared to support Th17/Treg balance to regulate inflammation by reducing *IL-6, IL-22* and *TGF-β1* as well as *CXCL-8* production while tending to decrease *NFKB1* expression. ZnO also suppressed *Reg3G*, an antimicrobial peptide and *GPX2,* a modulator of oxidative status, in jejunum. ZnO downregulated *TLR2, IL1B* and *IL-17A* while limiting *Reg3G*-induced epithelial responses in ileum, helping preserve gut integrity. Additionally, ZnO upregulated *PPARGC1A* (PPAR-*α*) to promote energy production in the ileum. *SLC39A4* was decreased in both tissues probably to limit excess zinc absorption. Overall, ZnO appears to support gut health through coordinated, site-specific transcriptional regulation of inflammation and gut barrier function pathways. PW, post-weaning (Figure created using draw.io).

## 1 Introduction

The supplementation of zinc oxide (ZnO) in the diets of weaned piglets has been a common strategy to mitigate post-weaning (PW) enteric infections, reduce the use of antibiotics, and enhance the growth performance of piglets (1–4). Weaning is a critical and stressful phase that is associated with reduced feed intake and impaired gut health, which is evidenced by gut inflammation, disrupted intestinal integrity, microbial imbalances, immunodepression and increased susceptibility to post-weaning diarrhea (PWD) (5–9). The use of pharmacological concentrations of ZnO (2,000–3,000 mg/kg) in postweaning feeds has been shown to improve growth performance of weanling pigs (1, 4, 10).

Zinc (Zn), commonly provided as ZnO, supports piglet gut health probably through several intertwined mechanisms to prevent PWD. It plays an essential role in numerous physiological processes including the synthesis of over 300 enzymes related to immune function and gut health (11–13). Firstly, ZnO at high doses may enhance intestinal barrier function, nutrient absorption, and intestinal development (14–16). ZnO supports the integrity of the gut barrier by promoting the activity of intestinal digestive enzymes and regenerating epithelial tissue (2, 17), upregulating the expression of tight junctions, such as *ZO-1* (18), and improving intestinal architecture, thereby promoting enterocyte proliferation (18, 19), enhancing nutrient absorption, and reducing gut permeability (14, 20, 21). Secondly, ZnO modulates intestinal immunity and redox balance (22), by suppressing pro-inflammatory cytokines and up-regulating anti-inflammatory factors (2, 13, 18). During infections, it reduces the expression of innate immunity related genes (23, 24), and alleviates oxidative stress induced by inflammatory processes (18, 25, 26). Finally, high ZnO doses also appear to modulate gut microbiota composition (27) by increasing alpha-diversity (28), similarly to certain growth-promoting antibiotics (15). In several studies, it has been shown that ZnO shifts the small intestinal microbiota towards non-pathological bacterial populations, contributing to a more stable intestinal environment during the PW period (6, 15, 27–29). A recent dose–response study revealed that ZnO inclusions above 2,400 mg/kg are required to markedly reshape the gut microbiota and modulate blood metabolic profile (30). These combined effects of ZnO on the piglets’ barrier integrity, digestive function, immune regulation, antioxidant defense, and microbial composition can explain the consistent reduction in PWD prevalence and improved performance (2, 11).

Zinc deficiency is known to affect all body organs, induce immune dysfunctions, and increase susceptibility to diseases (13). Conversely, the withdrawal of pharmacological doses of ZnO in pig diets may have detrimental effects on PW growth performance and gut health (31), including villi atrophy, reduced absorptive surface area and digestive enzyme secretion (32). During the immediate PW period, the piglets’ low feed intake may result in Zn deficiency, which could be overcome by the use pharmacological levels of ZnO in diet to help in meeting the animal’s Zn requirements (33). However, after a few days when feed intake is normalized, these high levels may become problematic as they might cause zinc overload (>2 ppm in serum) which has been associated with significant reductions in serum copper and selenium concentrations (34), as well as chronic pancreatitis (35). It has also been shown that pharmacological ZnO can increase the accumulation of Zn in the small intestine, and activate the expression of apoptosis-related genes, causing damage to the intestinal epithelium (36).

In addition, dietary zinc is poorly absorbed, and it accumulates in manure leading to soil contamination and toxicity concerns (11, 37, 38). Moreover, pharmacological doses of ZnO may interact negatively with other nutrients (38), promote the acquisition of antibiotic multi-resistant genes in zoonotic pathogens such as enterotoxigenic *Escherichia coli* (ETEC) (39), contributing to the emergence of antimicrobial resistances (AMR) and, finally, exacerbating public health concerns (33, 40, 41). Consequently, these concerns over environmental pollution, toxicity and AMR have prompted regulatory authorities to impose limits on permissible dietary zinc levels in pig production (11). For instance, the European Union has banned pharmacological ZnO use since 2022 and set the maximum allowable dietary zinc level for weaned piglets at 150 mg/kg (42). Hansen et al. (33) indicated that, at this concentration, newly weaned piglets with low voluntary feed intake cannot meet their zinc nutritional requirements, impairing gut barrier integrity and this may predispose the piglets to enteric infections (43). This could partially explain why piglets fed diets with 150 mg/kg of Zn are more susceptible to ETEC than those fed pharmacological levels of ZnO (44). In response to the ban, alternative strategies to pharmacological ZnO such as probiotics, prebiotics, organic acids, essential oils, spray dried porcine plasma have been explored (45–48), even though their efficacy in matching ZnO benefits has been inconsistent.

Even with the advancements in ZnO research, the mode of action of ZnO in the different regions of the intestine of piglets during the early post-weaning period is still not completely deciphered and its characterization remains critical for the future development of new alternatives (2, 45). To address this knowledge gap, we hypothesized that ZnO modulates the concentrations of key gut health biomarkers, and the expression of intestinal genes related to barrier function, immune response, oxidative stress, metabolic activity and digestive enzymes, to partially explain its mode of action. Therefore, we studied the effects of pharmacological doses of ZnO on selected gut health markers and expression of specific genes related to different gut functionalities in jejunum, ileum and caecum, aiming to contribute critical insights to the understanding of its mechanisms of action.

## 2 Materials and methods

This study adhered to the EU principles for the protection of animals used for scientific purposes and complied with Directive 2010/63/EU of 22 September 2010, as well as the Spanish regulations for the care and use of animals in research (Real Decreto 53/2013). The experimental procedures received approval from IRTA’s Ethical Committee for Animal Experimentation and the Commission on Animal Experimentation of Generalitat de Catalunya, Spain (Project number 11766).

### 2.1 Experimental design

The study was conducted in the postweaning facilities of the experimental farm of Animal Nutrition, IRTA (Centre Mas Bové, Constantí, Spain). A total of one hundred and eighty newly weaned piglets ([*Large White × Landrace*] × *Pietrain*; mixed sexes; 7.03 ± 1.12 kg BW) of around 26 days of age, in two separate batches (3 weeks apart) from IRTA’s experimental sow herd, were used. For each batch, the piglets were housed in two weaning rooms with 12 and 6 pens, respectively. The experimental herd had a relatively high-health status without clinical disease outbreaks having been recorded for at least the 12 months preceding the study. Therefore, the facilities were not cleaned or disinfected before the start of the trial with the intention of creating slightly challenging conditions for the animals. The rooms are provided with automatic heating, forced ventilation and completely slatted floors. Feed was distributed *ad libitum* in one feeding trough per pen with four feeding spaces. Water was also provided *ad libitum* throughout all the animals and experimental periods.

At the start of each batch, 90 piglets were selected and randomly distributed by initial body weight into nine blocks per batch. Each block consisted of two pens with five piglets each. There were two experimental treatments: Control diet and the same diet containing zinc oxide at 3,000 mg/kg (ZnO). Within each block, the two treatments were randomly distributed between pens according to a randomized complete block design.

At the start of each batch (day 0 PW) piglets were offered diets with pre-starter specifications until day 14. Feeds were formulated according to the minimum nutrient requirements as recommended by FEDNA (49) and the composition of the diets is presented in Table 1. The vitamin-mineral premix used in the basal diet contained 110 mg ZnO per kg of feed. For the ZnO diet, ZnO (0.3%) was added on top of the basal diet directly into the feed mixer. The feeds were presented in pelleted form and offered *ad libitum*.

**Table 1:** Ingredient composition and calculated nutrient contents of the basal diet and analyzed nutrient contents of the experimental diets (as-fed basis).

Ingredients, %	
Wheat	49.6
Soybean meal (44% CP)	29.9
Sweet milk whey	10.9
Sugar beet pulp	3.00
Animal fat	3.30
L-Lysine-HCl	0.33
DL-Methionine	0.08
L-Threonine	0.12
Sodium chloride	0.40
Calcium carbonate	0.07
Dicalcium phosphate	1.50
Formic acid	0.40
Vitamin-mineral premix*	0.40

Calculated nutrient composition, %	
Crude protein	21.5
Fat	4.63
Ash	5.88
Crude fiber	3.30
Metabolizable energy, kcal/kg	3285
SID Lysine	1.28
SID Methionine	0.39
SID Met+Cys	0.76
SID Threonine	0.83
SID Tryptophan	0.26
SID Valine	0.93
Total sodium	0.24
Total phosphorous	0.69
ATTD phosphorous	0.38
Total calcium	0.80

Analyzed nutrient composition, %	Control	ZnO
Dry matter	89.2	89.3
Crude protein	21.5	21.5
Fat	4.35	4.30
Ash	5.67	5.94
Crude fiber	3.03	3.06
Gross energy, kcal/kg	4071	4087
Total phosphorous	0.69	0.72
Total calcium	0.73	0.73

* Providing per kg feed: vitamin A (E-672) 10000 UI; vitamin D_3_ (E-671) 2000 UI; vitamin E (alfa-tocopherol) 25 mg; vitamin B_1_ 1.5 mg; vitamin B_2_ 3.5 mg; vitamin B_6_ 2.4 mg; vitamin B_12_ 20 μg; vitamin K_3_ 1.5 mg; calcium panthotenate 14 mg; nicotinic acid 20 mg; folic acid 0.5 mg; biotin 50 μg; Fe (E-1) (from FeSO_4_·H_2_O) 120 mg; I (E-2) (from Ca(I_2_O_3_)_2_) 0.75 mg; Cu (E-4) (from CuSO_4_·5H_2_O) 6 mg; Mn (E-5) (from MnO) 60 mg; Zn (E-6) (from ZnO) 110 mg; Se (E-8) (from Na_2_SeO_3_) 0.37 mg. CP, crude protein; ATTD, apparent total tract digestible; SID, standardized ileal digestible.

### 2.2 Performance and fecal scores

Feed (per pen) and individual piglets were weighed at days 0 and 14 PW. Pen’s average initial and final body weight, daily weight gain, feed intake and gain-to-feed ratio were calculated for the period 0–14 days. Fecal consistency was individually assessed using a 5-category score system (0 = firm and shaped; 1 = soft and shaped; 2 = soft without shape; 3 = loose; 4 = watery) as a slight modification of the scale proposed by Pederson and Toft (50). This was done by observing the feces in the pens; if they were firm and shaped, it was considered that all the piglets in that pen had a score of 0, if not, the piglets in that pen were individually scored by direct inspection. The highest individual scores given during weeks 1 and 2, respectively, were used for the statistical analysis.

### 2.3 Sample collection

At day 14 post-weaning, one animal per pen (the animal with intermediate initial body weight in each pen) was sampled for blood and euthanized to obtain samples of bile and intestinal mucosa. Blood serum and bile samples were collected into cryotubes and stored at −80 °C until analysis. Tissue fragments of 10 cm from jejunum (400 cm proximal from the ileo-cecal valve), ileum (50 cm proximal from the ileo-cecal valve) and caecum (5 cm distal from the ileo-cecal valve) were obtained and rinsed with saline solution, and the corresponding mucosa was scrapped and collected in 1 mL RNAlater (2 mL cryotube; 2 tubes per sample) before being frozen at −80 °C until analysis.

### 2.4 Gut barrier and immune indicators—ELISA

Commercial ELISA kits were employed, following the manufacturers’ protocols, for the quantitative measurement of calprotectin and citrulline in blood serum, and secretory immunoglobulin A (sIgA) in bile samples (CSB-EQ013485PI, Cusabio, China; K 6600, Immundiagnostik AG, Bensheim, Germany; and abx258080, Abbexa, Cambridge, United Kingdom, respectively). Absorbance was read at 450 nm using a microplate reader (Biotek Epoch2, Biotek Instruments, Winooski, Vermont, United States).

### 2.5 RNA extraction and reverse transcription

RNA was isolated from the jejunal, ileal, and cecal mucosa of the individual pigs using the miRNeasy Mini Kit (Qiagen, Hilden, Germany) according to the manufacturer’s protocol. The RNA concentrations and purities were assessed with a NanoDrop 1000 spectrophotometer (Thermo Scientific, Waltham, MA, United States). Extracted RNA samples were stored at −80 °C for future use. One μg of each RNA sample was used to synthesize complementary DNA (cDNA) using the PrimeScript RT Reagent Kit (Takara, RR037A, Dalian, China), following the instructions provided by the manufacturer.

### 2.6 Pre-amplification

Pooled cDNA samples were utilized to create a standard (STD) curve for quantification during qPCR. The pooled and individual cDNA samples diluted 1:5 in DNA suspension buffer (10 mM Tris–HCl, 1 mM EDTA, pH 8.0) underwent pre-amplification using the Preamp Master Mix (St Standard Biotools, San Francisco, CA, United States) and a custom primer mix containing 48 primer pairs at 500 nM each for subsequent qPCR analysis. The pre-amplification process included an initial denaturation at 95 °C for 10 min, followed by 15 cycles of 95 °C for 15 s and 60 °C for 4 min. Pre-amplification reactions were cleaned using Exonuclease I (Exo I) to remove residual primers. The pre-amplified cDNA -Exo I treated samples were subsequently diluted 1:20 using DNA suspension buffer prior to downstream analysis.

### 2.7 High-throughput gene expression analysis—qPCR

Quantitative real-time PCR (qPCR) was performed on the BioMark™ X9 system (Standard BioTools™, United States—formerly Fluidigm Corporation) utilizing 96.96 Dynamic Array Integrated Fluidic Circuit (IFC) chips. This setup allowed for 48 primer pairs in duplicates across 96 samples. The primers included in-house designs and modified versions of those previously reported by González-Solé et al. (51), sourced from Condalab (Spain), as detailed in Supplementary Table S1. This includes genes involved in: barrier function (*OCLN, CLDN15, ZO1, CLDN1, MUC2, MUC13,* and *CLDN4*), immune response, such as pattern recognition receptors, cytokines, chemokines and stress proteins (*pBD3, REG3G, CXCL8*—also referred as *IL8*, *IKBKB, IL6, TLR4, pBD2, GBP1, IFNG, IL22, TGFB1, TNFa, IFNGR1, TLR2, NFKBIA, DEFB1, IL10, IL17A, CCL20, NFKB1*, and *IL1B*), coding for enzymes and hormones implicated in the digestion process (*GPX2, DAO, PPARGC1A, IGF1R, IDO1, FAXDC2, SOD2, HNMT, CCK,* and *ALPI*), nutrient transport (*SLC5A1, SLC7A8, SLC16A1*, and *SLC39A4*), and stress response (*HSPA4* and *HSD11B1*); analyzed relative to reference genes (*GAPDH, ACTB, HPRT1* and *TBP*). Genes with minimal detection across samples were excluded from subsequent analyzes.

The qPCR sample mix was prepared by combining 2.7 μL of pre-amplified and Exo-I treated samples (diluted 1:20 DNA suspension buffer), 3.3 μL of 20 × EvaGreen DNA Binding Dye (Biotium, PN 31000), and 0.5 μL of 20 × DNA Binding Dye Sample Loading Reagent (Fluidigm, PN 100-0388). For each assay, the mix consisted of 27 μL of DNA suspension buffer, 30 μL of 2X Assay Loading Reagent and 3 μL of forward and reverse primers, each at a final concentration of 5 μM.

On the 96.96 Dynamic Array IFC chips, 5 μL of each sample mix and 5 μL of each assay mix were loaded into the corresponding inlets. Non-template controls (NTCs) to monitor potential contamination or nonspecific amplification and STD curve dilutions, prepared from pooled cDNA samples were included to the chip. The pre-amplified STD was diluted 1/5 with DNA suspension buffer and followed by five ¼ dilutions. The chips were processed on the BioMark™ X9 system under the following thermal cycling conditions: initial activation at 95 °C for 60 s, followed by 35 cycles of 96 °C for 5 s and 60 °C for 20 s. Fluorescence data were recorded at each cycle, and melting curve analyzes confirmed specific amplification.

Initial data quality control and threshold settings were performed using the BioMark™ system Real-Time PCR Analysis software (version 1.0.2; Standard BioTools, United States). Further normalization and efficiency corrections were conducted using DAG Expression software^1^ (52). Four porcine intestinal reference genes—GAPDH, ACTB, HPRT1, and TBP—were evaluated for stability, with TBP and GAPDH identified as the most stable reference genes for normalization.

### 2.8 Statistical analysis

All the performance parameters measured were compared between treatments by ANOVA using the PROC MIXED procedure of the statistical package SAS (SAS 9.4; SAS Institute Inc., Cary, NC, United States). A randomized complete block design was used with initial weight, batch and pen location as block criteria. For the statistical analysis, the block as a random factor and treatment as a fixed effect were included in the model and the pen was used as the experimental unit. Data in tables are presented as least-square means. Fecal consistency assessed using the maximum score given to each individual piglet each week. The score frequencies were analyzed using a Chi-square test of independence utilizing the PROC FREQ procedure in SAS software. ELISA data were Log transformed and tested for normality using the Shapiro–Wilk test, and then the differences between the Control and ZnO treatment groups were assessed using ANOVA in the GLIMMIX procedure of SAS software. The model included treatment as a fixed effect and block (same criteria as for performance) as a random effect. Differential gene expression values across the jejunum, ileum, and cecum were assessed using the GLIMMIX procedure in SAS. The model incorporated treatment as a fixed effect and block (same criteria as for performance) as a random effect. All the results were considered statistically significant at *p* < 0.05, while values of 0.05 ≤ *p* < 0.10 were interpreted as a tendency.

## 3 Results

### 3.1 Growth performance

The performance results (0–14 days) are shown in Table 2. Piglets from the ZnO group had higher final BW (*p* < 0.01) and average daily weight gain (ADG) than those from the control group (*p* < 0.01) and also tended to increase their average daily feed intake (ADFI) (*p* < 0.1). Moreover, ZnO improved the gain-to-feed ratio (GFR) compared to control (*p* < 0.05).

**Table 2:** Productive parameters of piglets fed control or ZnO diets between 0 and 14 days postweaning^a^.

Item	Control	ZnO	SEM	*p*-value
Initial weight (kg)	7.03	7.04	0.268	0.198
Final weight (kg)	8.75	9.36	0.423	**0.005**
Average daily gain (g/d)	123	165	31.2	**0.006**
Average daily feed intake (g/d)	181	204	23.8	0.063
Gain-to-feed ratio	0.669	0.774	0.085	**0.046**

a Values are presented as least squares means of 18 pens per treatment. ZnO, zinc oxide; SEM, standard error of the mean. Statistically significant *p*-values are highlighted in bold.

### 3.2 Fecal scores

**Figure 1:**
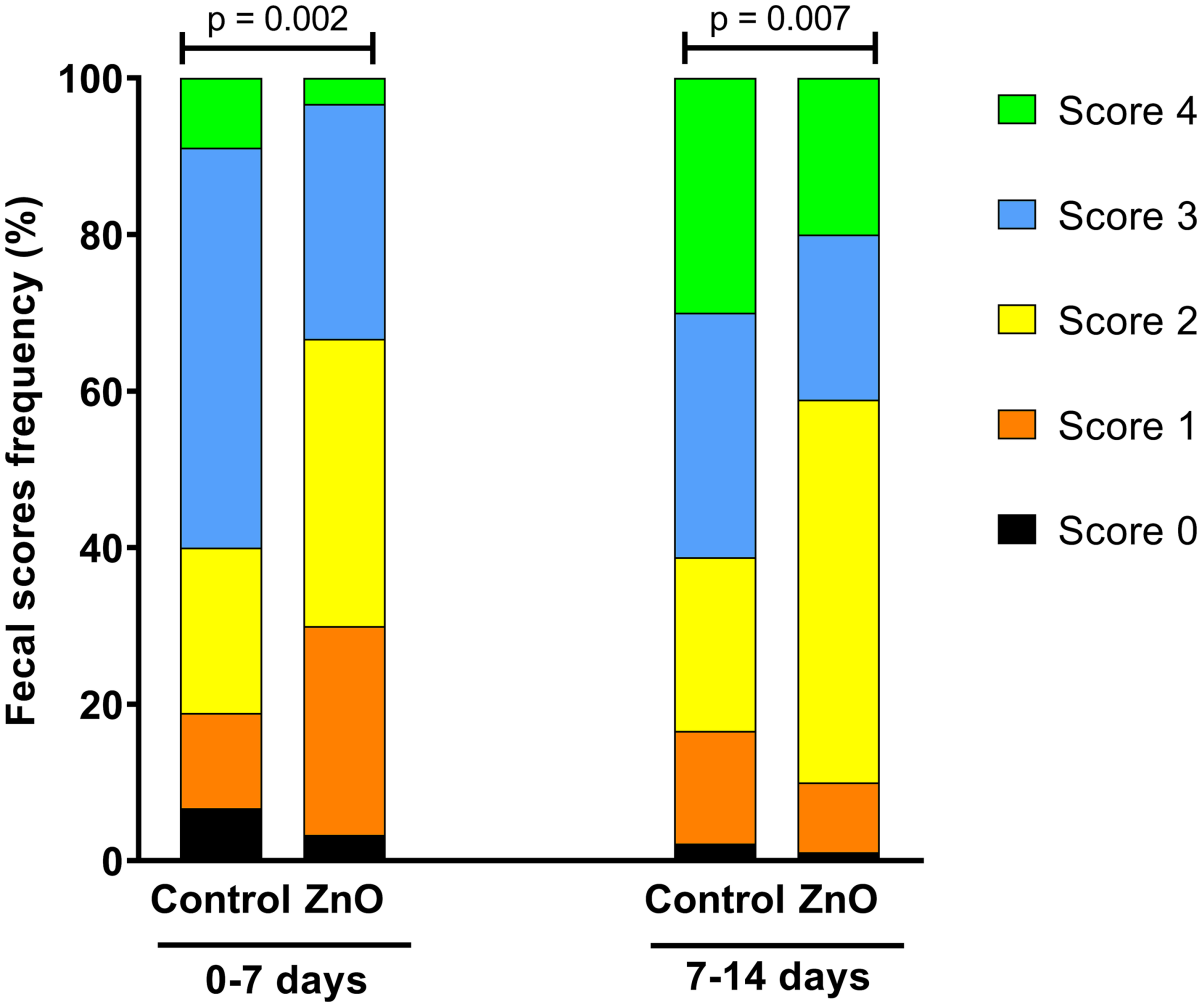
Fecal scores frequency (0 = firm and shaped; 1 = soft and shaped; 2 = soft without shape; 3 = loose; 4 = watery) of piglets between 0–7 days and 7–14 days postweaning.

The weekly fecal consistency results are shown in Figure 1. The analysis of individual fecal score frequencies revealed significant differences between treatments during week 1 (Chi-square = 16.82, *p* < 0.01) and week 2 (Chi-square = 14.05, *p* < 0.01). Notably, for the first week, 51% of the Control piglets had a fecal score of 3 (loose), compared to only 30% in the ZnO group. Additionally, 12% of the control pigs had a more consistent score of 1 (soft and shaped), compared to 27% of pigs in the ZnO group. During the second week, there were more piglets with loose (score of 3; Control = 31%, ZnO = 21%) and watery feces (score of 4; Control = 30%, ZnO = 20%) in the control group than in the ZnO group.

### 3.3 Biomarkers of gut health

The concentration levels of the intestinal health biomarkers analyzed in this study are shown in Table 3. Serum calprotectin levels, a marker of intestinal inflammation, were lower in ZnO-treated compared to control piglets (355 vs. 240 ng/mL, *p* < 0.01). Serum citrulline, as a biomarker of intestinal integrity, and bile sIgA, as an indicator of mucosal innate immunity, were unaffected by ZnO supplementation.

**Table 3:** Effects of ZnO on the Log-transformed concentrations of calprotectin and citrulline in blood serum and sIgA in bile at day 14 post-weaning^a^.

Item	Control	ZnO	*SEM*	*p-value*
Calprotectin (Log ng/ml)	2.55	2.38	0.055	**0.002**
Citrulline (Log μmol/l)	1.54	1.56	0.020	0.386
Secretory IgA (Log ng/ml)	3.19	3.08	0.101	0.405

a Values are presented as least squares means. SEM, standard error of the mean. Statistically significant *p*-values are highlighted in bold.

### 3.4 Intestinal gene expression profile

Gene expression analysis using microfluidic qPCR provided insights into the molecular effects of ZnO on gut health. All the measured relative gene expression values in jejunum, ileum and cecum are shown in Table 4.

**Table 4:** Relative gene expression in the piglets’ jejunal, ileal and cecal mucosa of control and ZnO treatment relative to the reference genes at day 14 post-weaning^a^.

Item		*Jejunum*				*Ileum*				*Caecum*			
Function	Gene	Control	ZnO	*SEM*	*p-value*	Control	ZnO	*SEM*	*p-value*	Control	ZnO	*SEM*	*p-value*
BF	*OCLN*	1.51	1.37	0.136	0.431	1.99	2.02	0.210	0.886	1.14	1.49	0.223	0.261
	*CLDN15*	4.55	5.97	1.804	0.539	6.88	2.52	1.654	0.073	1.58	2.26	0.354	0.183
	*ZO1*	1.59	1.55	0.222	0.888	1.24	1.00	0.129	0.208	0.61	0.57	0.044	0.473
	*CLDN1*	8.39	9.75	3.509	0.766	12.69	6.01	2.991	0.112	5.17	4.52	1.229	0.456
	*MUC2*	1.56	1.09	0.175	0.076	0.78	0.55	0.102	0.073	0.66	0.73	0.066	0.500
	*MUC13*	0.77	0.72	0.089	0.650	0.54	0.60	0.085	0.410	0.45	0.43	0.084	0.831
	*CLDN4*	2.52	1.76	0.592	0.187	2.54	2.13	0.392	0.476	2.96	2.63	0.667	0.734
IR	*pBD3*	2.12	3.17	0.689	0.241	0.95	0.76	0.150	0.399	0.57	0.90	0.152	0.080
	*REG3G*	2.54	0.19	0.558	**0.008**	1.01	0.12	0.166	**0.001**	0.65	0.90	0.318	0.419
	*CXCL8 (IL8)*	1.95	1.05	0.249	**0.020**	0.83	0.48	0.134	0.060	1.61	1.18	0.223	0.138
	*IKBKB*	3.82	3.69	0.450	0.819	3.65	2.82	0.576	0.324	–	–	–	–
	*IL6*	1.45	0.61	0.223	**0.015**	0.62	0.64	0.073	0.803	1.37	1.14	0.160	0.268
	*TLR4*	1.58	1.00	0.311	0.179	0.62	0.57	0.072	0.631	0.96	0.82	0.142	0.441
	*pBD2*	0.84	0.73	0.121	0.545	0.91	0.77	0.141	0.340	0.44	0.47	0.091	0.805
	*GBP1*	2.27	2.12	0.276	0.623	2.38	2.28	0.320	0.784	1.59	1.55	0.222	0.910
	*IFNγ*	2.48	1.65	0.467	0.055	4.45	3.70	0.729	0.304	–	–	–	–
	*IL22*	1.69	0.30	0.464	**0.045**	0.38	0.15	0.101	0.137	0.78	0.74	0.187	0.825
	*TGF-β1*	1.85	1.34	0.114	**0.002**	1.48	1.42	0.130	0.722	2.31	2.11	0.212	0.435
	*TNFa*	2.24	2.11	0.321	0.778	1.16	1.22	0.159	0.796	3.14	2.75	0.294	0.273
	*IFNGR1*	0.87	0.78	0.097	0.499	0.81	0.77	0.136	0.716	0.56	0.56	0.068	0.975
	*TLR2*	2.32	1.73	0.462	0.287	1.03	0.62	0.125	**0.031**	0.75	1.06	0.125	**0.023**
	*NFκBIA*	2.29	2.39	0.447	0.842	2.70	1.72	0.496	0.178	0.00	0.00	0.002	0.315
	*DEFB1*	0.88	0.88	0.170	0.981	0.67	0.61	0.141	0.771	2.19	1.66	0.481	0.439
	*IL10*	1.89	1.52	0.175	0.095	0.73	0.86	0.106	0.360	1.11	1.26	0.137	0.406
	*IL17A*	3.57	1.79	0.784	0.115	0.41	0.11	0.086	**0.033**	1.32	0.90	0.305	0.321
	*CCL20*	1.07	1.49	0.266	0.276	0.26	0.20	0.056	0.453	3.20	2.14	0.572	0.155
	*NFκB1*	1.77	1.58	0.082	0.081	0.91	0.91	0.039	0.929	–	–	–	–
	*IL1β*	1.53	0.81	0.305	0.112	0.62	0.24	0.112	**0.029**	1.44	1.28	0.366	0.781
MEHA	*GPX2*	2.38	1.18	0.324	**0.005**	1.16	0.71	0.189	0.087	0.58	0.66	0.087	0.479
	*DAO*	0.98	1.09	0.181	0.651	1.66	1.64	0.434	0.965	0.34	0.61	0.184	0.314
	*PPARGC1A*	1.39	1.67	0.163	0.225	1.09	1.57	0.161	**0.021**	0.83	1.15	0.166	0.193
	*IGF1R*	1.99	1.68	0.177	0.159	1.09	1.19	0.117	0.535	0.88	0.92	0.090	0.662
	*IDO1*	1.53	0.82	0.433	0.146	0.98	0.90	0.183	0.625	2.81	1.49	0.573	0.122
	*FAXDC2*	2.77	2.77	0.402	1.000	4.79	2.86	0.859	0.071	1.53	1.13	0.376	0.388
	*SOD2*	1.58	1.37	0.184	0.349	1.20	1.03	0.141	0.256	1.01	0.85	0.093	0.123
	*HNMT*	1.00	1.00	0.066	0.965	1.49	1.40	0.119	0.425	0.95	0.80	0.129	0.354
	*CCK*	1.42	1.41	0.140	0.954	0.73	0.85	0.134	0.372	5.66	4.73	1.280	0.400
	*ALPI*	1.35	1.59	0.200	0.209	1.19	1.12	0.159	0.699	3.54	1.02	1.121	0.093
NT	*SLC5A1*	1.19	1.17	0.269	0.950	0.97	0.83	0.266	0.617	0.87	0.72	0.143	0.381
	*SLC7A8*	1.21	1.13	0.349	0.868	0.50	0.45	0.054	0.466	1.27	1.34	0.158	0.678
	*SLC16A1*	0.84	0.65	0.101	0.186	0.78	0.78	0.130	0.989	0.23	0.35	0.085	0.141
	*SLC39A4*	2.20	0.53	0.269	**<0.001**	2.16	0.56	0.331	**0.002**	0.82	0.71	0.071	0.224
SI	*HSPA4*	0.80	0.71	0.045	0.154	0.81	0.84	0.070	0.792	0.70	0.70	0.071	0.962
	*HSD11B1*	2.26	2.69	0.536	0.528	1.07	0.94	0.249	0.651	1.28	0.86	0.206	0.078

a Values are presented as least squares means [Normalized quantified expressions (NormQ)]. SEM, standard error of the mean. Statistically significant *p*-values are highlighted in bold. Functions: MEHA, metabolic, enzymatic and hormonal activity; IR, immune response; NT, nutrient transport; SI, stress indicators; BF, barrier function. For list of gene names see Supplementary Table S1.

In Jejunum, several genes showed differential expression between the control and ZnO treatments. The expression of the immune-related genes *REG3G*, *CXCL8/IL8*, *IL-6*, *IL-22*, and *TGFB1* was downregulated in the ZnO group compared to the control group (*p* < 0.05). The expressions of the Zn transporter gene *SLC39A4* and the antioxidative-related gene *GPX2* were also decreased in the ZnO fed piglets (*p* < 0.01). Finally, ZnO also tended to downregulate the expressions of *IFNγ*, *MUC2*, *IL-10*, and *NFκB1* (*p* < 0.1).

In the ileum, ZnO downregulated pro-inflammatory and other immune-related genes at d 14 PW, such as *REG3G*, *IL-17A*, *IL-1B*, and *TLR2* (*p* < 0.05). The expression of *SLC39A4* was also downregulated in the ileum (*p* < 0.001) of ZnO fed piglets. The only gene that was upregulated by ZnO in the ileum was *PPARGC1A* (*p* < 0.05). Moreover, tendencies for the downregulation of the gene expressions for *CLDN15, MUC2, CXCL8/IL-8, GPX2*, and *FAXDC2* were also observed in the ileum of the ZnO group (*p* < 0.1).

In the caecum, *TLR2* gene was upregulated in ZnO fed piglets compared to the control (*p* < 0.05).

## 4 Discussion

Weaning is a critical phase in swine production in which environmental and pathogenic stressors are often linked to impairment of gut health, high incidence of PWD, reduced performance and increased mortality (5, 9). Dietary ZnO at high doses of up to 3,000 mg/kg has been widely used in pre-starter diets for weaned pigs due to its potent antimicrobial and growth performance enhancing capacities (2, 18). However, the regulatory ban on this use of ZnO in Europe, driven by concerns over Zn environmental pollution and antimicrobial resistances emergence among others, has generated the need to develop sustainable effective alternatives to ZnO, for which a deeper understanding of its mechanisms of action are required (11, 42). With the aim to better understand the modes of action of ZnO in weaned pigs, the present study investigated the effects of high levels of ZnO supplementation on growth performance, gut health biomarkers in blood and bile, and the gene expression in different segments of the intestine for key genes related to barrier function, immune response, metabolic and enzymatic activity, stress, and nutrient transport.

In the current trial, we observed that high doses of ZnO increased ADG, ADFI, and feed efficiency, results that are consistent with many previous studies (1, 10, 53). Hollis et al. (54) demonstrated that pigs fed 2,000 mg/kg ZnO gained weight faster and consumed more feed than those fed either 500 mg/kg ZnO or organic Zn sources. Similarly, Hill et al. (10) observed that supplemental ZnO at 1,500 to 2,000 mg Zn/kg improved postweaning pig performance, and in another study the supplementation with 3000 mg/kg ZnO increased BW after 14 d PW (18).

These performance benefits are often attributed to the ability of ZnO to reduce the incidence of PWD (15, 55). In our study, the piglets from the ZnO group had better fecal consistency, indicative of a lower prevalence of diarrhea, underscoring the efficacy of ZnO to manage post-weaning challenges. This is in line with numerous studies that have reported similar results in weaned pigs (18, 20, 56). These improvements are likely mediated by antimicrobial properties of ZnO, which can inhibit the adhesion of pathogenic bacteria to intestinal cells and prevent the subsequent disruption of intestinal integrity (11, 21). Direct effects of ZnO on pathogens have not been completely defined, although electrostatic forces, production of Zn^2+^ ions and the generation of reactive oxygen species have been described as possible pathways of the bactericidal action of ZnO (57). Recent research proposes that the mechanism of action of ZnO involves the reduction of toxic and inflammatory microbial metabolites resulting from a lower abundance of pathogenic bacteria in the gut (30, 58).

A key finding of our study was the reduction of serum calprotectin levels in the piglets fed ZnO. Calprotectin, an innate immune protein complex with antimicrobial activity, can bind Zn and other metals with high affinity, and starves bacteria of these essential nutrients (59, 60). As a marker of inflammation that is released by activated neutrophils, calprotectin in serum is elevated in conditions of gut inflammation and barrier dysfunction in different species (61, 62). In the current study, high levels of dietary ZnO may have directly prevented neutrophil activation and subsequent calprotectin release triggered by the recognition of bacterial pathogens. Moreover, it has been shown in mouse jejunal organoids that calprotectin increases the expression of gene *SLC39A4* which encodes for the Zn transporter ZIP4 (63). This transporter can modulate the intracellular and extracellular concentrations and availability of Zn^2+^ in response to available Zn^2+^ levels (63). In our study we observed a clear downregulation of *SLC39A4* expression in both jejunum and ileum of the ZnO piglets, which might be explained by the reduction of gut inflammation (less calprotectin release), or because the high circulant Zn^2+^ available makes unnecessary the overexpression of Zn transporters for Zn^2+^ cell internalization. ZIP4 is important for Zn homeostasis and absorption from the small intestine, as well as for the regulation of intestinal epithelial function (64, 65); it can therefore be indicative of Zn absorption and utilization (66). Similarly to what we have observed in both the jejunum and the ileum, high doses of Zn lead to the downregulation of *SLC39A4* in the jejunum of piglets (67, 68). Interestingly, the expression of ZIP4 is regulated by Zn, being increased in conditions of deficiency and decreased with adequate levels (69, 70). This suggests an adequate Zn status with the high ZnO diets, limiting further uptake and the possibly of mitigating Zn induced cytotoxicity (71). Alternatively, it may also reflect that the dose of Zn administered exceeded the animals’ physiological needs, suggesting that such high levels may not be necessary to meet the nutritional requirements of healthy piglets.

Serum citrulline, a biomarker for intestinal enterocyte mass and function (72), was unaffected by dietary ZnO at day 14 PW. This is in line with longitudinal studies showing that citrullinemia falls only during the first 7 to 10 days after weaning and returns to baseline levels by day 15, thus making it less sensitive to the inflammatory changes relieved by ZnO in the current study (73–75). Secretory IgA provides the first line of innate mucosal defense by neutralizing luminal pathogens without provoking inflammation (76). Although most of the sIgA in the gut lumen is released locally, a proportion is delivered into the upper intestine via the biliary tract (77–80), and biliary sIgA has been used to assess mucosal immune status in pigs (81, 82). We found that pharmacological ZnO had no effect on biliary sIgA levels at 14 d PW. However, Ramis et al. (83) observed reduced levels of fecal sIgA with ZnO supplementation at 8 d PW but that was not observed at later stages (11–15 d PW). Although the lack of differences in the current study might be attributed to the limitations of using bile instead of intestinal contents to measure sIgA, it could also be due to the temporal dynamics of the immune response to ZnO.

During the PW period, piglets experience both prolonged and transient changes of gene expression of inflammatory cytokines like *IFN-γ, IL-1β, IL-6, IL-8, IL-10, IL-12α,* and *TGF-β* in jejunum, ileum, and colon (84). Our study revealed that ZnO reduces the expression of inflammatory genes in the small intestine. Notably, ZnO downregulated *REG3G*, *IL-6, IL-8, IL-22*, and *TGF-β1* in the jejunum and *IL-1β, REG3G, TLR2*, and *IL-17A* in the ileum. Contrarily, ZnO increased *TLR2* in the cecum. These results align with previous studies where ZnO prevented the induction of inflammatory processes, pointing out to a coordinated effort to maintain gut homeostasis in the PW period (18, 23, 24, 85). Our observation that ZnO downregulated jejunal *IL-6,* and *IL-8, and* tended to downregulate that of *IFNγ* and *NFκB1*, supports its proposed capacity to modulate pro-inflammatory signaling (86). Consistent with our findings in jejunum, Hu et al. (87) observed decreased mucosal mRNA levels of *IL-6* and *IFN-γ* at day 7 PW using diosmectite ZnO at 500 mg/kg in early weaned pigs. Thus, ZnO may contribute to preserve epithelial integrity by suppressing the expression of proinflammatory cytokines which can lead to increased intestinal permeability (88).

Interestingly, in ZnO piglets at day 14, the gene expression for cytokines *TGF-β1* and *IL-22* was downregulated in jejunum, and that of *IL-17A* in ileum; cytokines that are important key regulators for the epithelial barrier function and repair (89, 90). These results contrast with previous findings showing that ZnO increased *TGF-β* expression in the intestine of weaned piglets at 8 d PW (91) and jejunal mucosa at 28 d PW (18), suggesting a role in repair. Our data suggest that in a context of overall reduced inflammation, the stimuli for *TGF-β1* production are also reduced, pointing to a state of quiet homeostasis rather than active repair. This discrepancy may be explained by the pleiotropic nature of *TGF-β1*, which, together with *IL-6*, contributes to the regulatory signaling of T helper 17 (Th17) cells and regulatory T-cells (Tregs) (92–94). Zn is a known modulator of this axis, promoting the development of anti-inflammatory Treg cells while suppressing the differentiation of pro-inflammatory Th17 cells (95). Th17 cells can also promote tissue repair and homeostasis, an effect mediated by *IL-22* by promoting the regeneration of epithelial tissues (96). Specifically, ZnO has been shown to directly inhibit phosphorylation of STAT3, a key transcription factor required for Th17 differentiation, thereby reducing the production of Th17-associated cytokines like *IL-17A* and *IL-22* (97). Taken together, the downregulation of *IL-22* in jejunum and *IL-17A* in ileum in ZnO-fed piglets is consistent with the lower serum calprotectin concentrations observed, because both cytokines can induce calprotectin expression in epithelial cells (98). This could be a possible mode of action of ZnO to dampen a major pathway of intestinal inflammation in jejunum. However, these observations require other functional assays to validate these potential mechanisms.

The gene *REG3G,* which encodes for an important C-type lectin that has bactericidal activity against Gram-positive bacteria, is induced by *IL-22* via STAT3 pathway activation (99). Notably, we observed decreased expression of *REG3G* in both jejunum and ileum, despite the expression of *IL-22* only being reduced in jejunum. Interestingly, Schokker et al. (28) also observed *REG3G* downregulation in both tissues from clinically healthy piglets fed with high doses of ZnO. This gene is also a regulator of Th17 cell mediated immunity as has been observed in mice, playing an important role in the physical segregation of microbiota from the host as well as in the immune response induced by pulmonary pathogens (100). All these findings support the hypothesis that ZnO suppresses inflammatory processes and consequently, Th17 responses, which aligns with the mechanistic evidence provided by Kitabayashi et al. (97), who showed that zinc directly inhibits Th17 cell development by attenuating STAT3 activation.

Beyond immunomodulation, ZnO has been shown to enhance the redox balance and prevent apoptosis in the jejunum of piglets at 15 d PW, effectively reducing weaning-related intestinal dysfunction and nutrient malabsorption (25). In our study, the jejunal downregulation of *GPX2*, a key antioxidant enzyme, suggests that ZnO may mitigate oxidative stress to protect enterocytes from damage, which is consistent with the reported decrease of plasma malondialdehyde levels, an indicator of oxidative stress or lipid peroxidation, in ZnO fed piglets (18).

In the ileum, ZnO downregulated the expression of *IL-1β* and *TLR2* and tended to downregulate that of *IL-8* and *FAXDC2*. The reduced *IL-1β* expression in our study is consistent with the results of Zhu et al. (18) who observed a downregulation of *IL-1β* expression in the jejunal mucosa by ZnO signifying its role in dampening inflammation. The observed trend for gene expression downregulation of *IL-8* in our study, which is involved in neutrophil recruitment, might be linked to its regulation by the TLR2/MyD88/NF-κB signaling pathway (101). Although the influence of Zn on this pathway is not well established, other studies have shown that ZnO supplementation downregulates *TLR2* and *TLR4* expression (102) and influences MAPK signaling pathway in weaned piglets (91). We observed a *TLR2* downregulation in the ileum of ZnO-fed piglets, which is noteworthy considering that *TLR2* mRNA expression is naturally high in the Peyer’s patches of the ileum (103). The suppression of *TLR2* expression in ileum by ZnO may consequently attenuate NF-κB activation, thereby reducing the production of pro-inflammatory cytokines (85, 104). As it is known that Zn deficiency activates NF-κB and MAPK signaling (105), our findings reinforce the immunoregulatory role of ZnO in supporting both innate and adaptive immunity, helping to alleviate intestinal inflammation and enhance gut defense mechanisms in weaned piglets.

In the cecum, *TLR2* was upregulated in ZnO fed piglets, suggesting an enhanced state of immune readiness in the hindgut. *TLR2* triggers inflammatory and immune reactions by recognizing diverse microbial components (106). This expression in the cecum may be driven by the dense and diverse local microbiota, which provides potent *TLR2* ligands like peptidoglycan and lipoproteins and determines the region-specific immune status (106–108). In fact, expression patterns of intestinal TLRs differ significantly across gut segments, with microbiota composition and physiological events such as weaning influencing markedly higher expression in the colon and cecum (109–111). This might explain our observed downregulation in the ileum compared to the upregulation in cecum with the use of ZnO. Considering that ZnO is known to alter gut microbiota, the amplified *TLR2* signaling in the cecum may therefore reflect the site-specific immunomodulation driven by diet-induced microbial changes, thereby potentially contributing to improved mucosal barrier integrity, gut health and PWD management (102, 112, 113).

The gene *PPARGC1A,* also known as *PGC-1α*, was the only upregulated gene in the ileum of ZnO supplemented piglets. *PPARGC1A* encodes a transcriptional coactivator that interacts with *PPARγ*, regulating genes involved in lipid metabolism, ATP production, and glucose homeostasis, while also suppressing inflammation through negative crosstalk with the NF-κB signaling pathway (114, 115). Supporting this role, Handschin et al. (116), showed that mice lacking *PGC-1α* in skeletal muscle exhibited elevated circulating IL-6 and impaired glucose regulation, while Pérez et al. (117), demonstrated that *PGC-1α* deficiency in the pancreas amplified NF-κB-mediated *IL-6* expression and worsened inflammation during pancreatitis. In our study, we observed upregulation of *PPARGC1A* in the ileum and downregulation of *IL-6* expression in the jejunum of ZnO-supplemented piglets, suggesting a broader anti-inflammatory effect of ZnO, potentially mediated through PGC-1α–dependent suppression of NF-κB and *IL-6* signaling. This is supported by evidence that PPAR signaling requires Zn to exert its anti-inflammatory effects (115). Previous studies have shown that Zn increased the mRNA expression of *PPARγ* in weaned piglets (118), while Zn deficiency decreased *PPARγ* signaling in porcine endothelial cells (119). In a study by Bao et al. (114), Zn increased *PPARGC1A* expression in the ileum of elderly humans, while weaning in piglets has been shown to reduce the expression of *PGC-1α* (120). The current study, therefore, offers key evidence that ZnO may support *PPARγ* signaling, contributing to its anti-inflammatory and antioxidant actions, likely through NF-κB inhibition.

Collectively, these findings support the reported immunomodulatory and antimicrobial role of ZnO by modulating innate immunity, probably by reducing pathogenic bacterial loads and stabilizing microbiota consistently across the small intestine (2, 21, 121). From our findings, it could be speculated that ZnO dampens inflammatory responses across the small intestine by inhibiting the NF-κB signaling pathway and the Th17 response through STAT3 inactivation, as suggested by the reduced expression of *REG3G*, to achieve gut homeostasis in a multi-faceted way. The inhibition of NF-κB by Zn via TLR, calprotectin and *PPARγ* signaling pathways is therefore a possible mechanism through which Zn attenuates pro-inflammatory processes and mitigates oxidative stress. The pronounced modulation observed in jejunum, followed by some similar but lower influences in ileum, with minimal changes in the caecum, may be attributed to their critical functions as the primary segments exposed to dietary changes after weaning, making them more susceptible to ZnO. Importantly, our study provides novel intestinal segment-specific molecular insights that can be used when evaluating potential alternatives to ZnO, with the objective of replicating its beneficial effects on gut health. We also note the need to maintain a delicate balance, where host immune cells require an adequate Zn supply to sustain an effective immune response against stressors, while simultaneously restricting Zn availability to pathogens and toxicity risks.

However, we acknowledge that a key limitation of our study is that protein levels and activity were not evaluated to establish causality. While transcriptomic analysis provides valuable insight into potential mechanisms, mRNA levels do not always translate directly to abundance or functional state of proteins due to complex post-transcriptional and post-translational regulation. Therefore, future research is warranted to validate our segment-specific findings at the protein level using targeted proteomic and functional assays.

## 5 Conclusion

The understanding of the molecular mechanisms by which ZnO preserves gut health is essential for the identification of pharmacological targets and the development of alternatives. Pharmacological levels of ZnO improved performance and fecal consistency in weaned piglets. Additionally, ZnO modulated the expression of genes related to the maintenance of intestinal health, mainly in the ileum and the jejunum. These include the suppression of oxidative stress (*GPX2*) and the modulation of gut microbiota (*REG3G*) and innate immunity (*TLR2*). ZnO reduced the gene expression of pro- and anti-inflammatory cytokines (*IL8*, *IL6*, *IL22*, *IL17A*, *IL1B*, and *TGFB1*), reduced Zn transport and absorption (*SLC39A4*), enhanced mitochondrial function (*PPARGC1A*), and attenuated neutrophil-mediated inflammation (serum calprotectin). These findings are indicative of the roles of ZnO to regulate different pathways such as NF-κB, STAT3, Th17/Treg balance, and PPARγ signaling pathway. Collectively, the performance benefits of dietary supplementation with high doses of ZnO in weaning piglets may be explained through the maintenance of intestinal homeostasis, particularly that of the gut immune function, resulting from the anti-inflammatory, antioxidant, and antimicrobial properties of ZnO.

## Data Availability

The datasets used and/or analyzed to support the findings of this study are available from the corresponding author on a reasonable request.

## Footnotes

^1^ www.dagexpression.com
