# Supplementary material for: Impact of zinc oxide on gut health, immunity, and growth in weaned piglets: exploring potential modes of action
Source: Front Vet Sci. 2025 Sep 1;12:1645900. doi: 10.3389/fvets.2025.1645900 (PMC12434765; doi:10.3389/fvets.2025.1645900)
Supplement: Supplementary file 1 [file Table_1.docx]

Supplementary Material

# Supplementary Table

## Table S1. List and details of primers used in the high throughput gene expression study.

| *Function* | *Gene* | *Name* | *5'->3'* | *Primer Sequence* | *Accession number* | *Tm* | *GC%* | *Amplicon length* |
| --- | --- | --- | --- | --- | --- | --- | --- | --- |
| Permeability and Intestinal Barrier | *OCLN* | Occludin | Forward | CAGGTGCACCCTCCAGATTG | NM_001163647.2 | 60.68 | 60.00 | 126 |
|  |  |  | Reverse | AGGCCTATAAGGAGGTGGACTT |  | 60.02 | 50.00 |  |
|  | *ZO1* | Zonula occludens-1 | Forward | GGCTATGTCCAGAATCTCGGAAAA | XM_021098856.1 | 60.68 | 45.83 | 143 |
|  |  |  | Reverse | TGCTTCTTTCAATGCTCCATACC |  | 59.30 | 43.48 |  |
|  | *CLDN1* | Claudin-1 | Forward | ACCCCAGTCAATGCCAGATATG | NM_001244539.1 | 60.16 | 50.00 | 91 |
|  |  |  | Reverse | AAAGTAGGGCACCTCCCAGAAG |  | 61.69 | 54.55 |  |
|  | *CLDN4* | Claudin-4 | Forward | CCTCCGTGCTGTTCCTCAA | XM_005661969.2 | 59.63 | 57.89 | 83 |
|  |  |  | Reverse | GAGGCACAAGCCCAGCAA |  | 60.60 | 61.11 |  |
|  | *CLDN15* | Claudin-15 | Forward | GGATGGTGGCTATCTCCTGGTA | NM_001161643.1 | 60.76 | 54.55 | 88 |
|  |  |  | Reverse | GGGCCCAGCTCATACTTGGT |  | 61.93 | 60.00 |  |
|  | *MUC2* | Mucin 2 | Forward | GGACGACACCATCTACCTCACT | XM_021082584.1 | 61.21 | 54.55 | 131 |
|  |  |  | Reverse | GGCCAGCTCGGGAATAGAC |  | 59.93 | 63.16 |  |
|  | *MUC13* | Mucin 13 | Forward | CAGTGGAGTTGGCTGTGAAAAC | NM_001105293.1 | 60.22 | 50.00 | 150 |
|  |  |  | Reverse | ATCAAGTTCTGTTCTTCCACATTCTTG |  | 60.26 | 37.04 |  |
| Oxidative state | *GPX2* | Glutathione peroxidase 2 | Forward | GCAACCAATTTGGACATCAGGAG | NM_001115136.1 | 60.37 | 47.83 | 99 |
|  |  |  | Reverse | GGGTAAAGGTGGGCTGGAAT |  | 59.66 | 55.00 |  |
|  | *SOD2* | Superoxide dismutase | Forward | GGGGTTGGCTCGGTTTCAA | NM_214127.2 | 60.53 | 57.89 | 123 |
|  |  |  | Reverse | CATGCTCCCACACGTCGAT |  | 60.15 | 57.89 |  |
| Digestion and Metabolism | *ALPI* | Intestinal alkaline phosphatase | Forward | ATGTCTTCTCTTTTGGTGGCTACA | XM_003133729.4 | 60.20 | 41.67 | 92 |
|  |  |  | Reverse | GGAGGTATATGGCTTGAGATCCA |  | 59.16 | 47.83 |  |
|  | *DAO* | D-amino acid oxidase | Forward | GAACCAACAGACCTTCAACTATCTC | NM_214066.2 | 59.36 | 44.00 | 148 |
|  |  |  | Reverse | CTTTCGGAATCCCAGGACCAT |  | 59.79 | 52.38 |  |
|  | *HNMT* | Histamine N-methyltransferase | Forward | TGTTGAACCAAGTGCTGAACAAAT | NM_001244561.1 | 60.08 | 37.50 | 76 |
|  |  |  | Reverse | CTTTATGTTCTCGAGGTTTGATGTCTT |  | 59.88 | 37.04 |  |
|  | *IDO1* | Indoleamine 2,3 dioxygenase | Forward | GTCTTGGCAAATTGGAAGAAAAAGG | NM_001246240.1 | 59.76 | 40.00 | 80 |
|  |  |  | Reverse | CCCGGAAATGAGAAGAGAATATCCAT |  | 60.52 | 42.31 |  |
|  | *CCK* | Cholecystokinin | Forward | CAGCAGGCTCGAAAAGCAC | NM_214237.2 | 59.79 | 57.89 | 109 |
|  |  |  | Reverse | AATCCATCCAGCCCATGTAGTC |  | 59.89 | 50.00 |  |
|  | *IGF1R* | Insulin-like growth factor 1 receptor | Forward | CCGACGCGGCAACAAC | NM_214172.1 | 59.40 | 68.75 | 116 |
|  |  |  | Reverse | TCAGGAAGGACAAGGAGACCAA |  | 60.70 | 50.00 |  |
|  | *PPARGC1A* | Peroxisome proliferative activated receptor gamma, coactivator 1 alpha | Forward | CTCTGGAACTGCAGGCCTAA | NM_213963.2 | 59.38 | 55.00 | 79 |
|  |  |  | Reverse | TGGAGAAGCCCTAAAAGGGTTAT |  | 59.14 | 43.48 |  |
| Immune Response | *IL1B* | Interleukin 1 beta | Forward | GGTGACAACAATAATGACCTGTTATTTG | NM_214055.1 | 59.71 | 35.71 | 99 |
|  |  |  | Reverse | GCTCCCATTTCTCAGAGAACCA |  | 60.03 | 50.00 |  |
|  | *IL6* | Interleukin 6 | Forward | TCCAATCTGGGTTCAATCAGGAG | NM_214399.1 | 60.05 | 47.83 | 124 |
|  |  |  | Reverse | ACAGCCTCGACATTTCCCTTATT |  | 60.05 | 43.48 |  |
|  | *CXCL8* | Interleukin 8/C-X-C motif chemokine ligand 8 | Forward | GGAAAAGTGGGTGCAGAAGGT | NM_213867.1 | 60.75 | 52.38 | 96 |
|  |  |  | Reverse | GGAGAATGGGTTTTTGCTTGTTGT |  | 60.68 | 41.67 |  |
|  | *IL10* | Interleukin 10 | Forward | GAGGCTGCGGCGCT | NM_214041.1 | 59.30 | 78.57 | 84 |
|  |  |  | Reverse | AGCTTGCTAAAGGCACTCTTCA |  | 60.22 | 45.45 |  |
|  | *IL22* | Interleukin 22 | Forward | TGTTCCCCAACTCTGATAGATTCC | XM_021091968.1 | 59.84 | 45.83 | 131 |
|  |  |  | Reverse | GTTGTTCACATTTCTCTGGATATGCT |  | 59.91 | 38.46 |  |
|  | *IL17A* | Interleukin 17A | Forward | CCAGACGGCCCTCAGATTAC | NM_001005729.1 | 59.89 | 60.00 | 144 |
|  |  |  | Reverse | GATCTTCCTTCCCTTCAGCATTG |  | 59.37 | 47.83 |  |
|  | *TLR2* | Toll-like receptor 2 | Forward | CTCTCGTTGCGGCTTCCA | NM_213761.1 | 60.05 | 61.11 | 115 |
|  |  |  | Reverse | AAGACCCATGCTGTCCACAAA |  | 60.13 | 47.62 |  |
|  | *TLR4* | Toll-like receptor 4 | Forward | CATCCCCACATCAGTCAAGATACT | NM_001113039.2 | 59.90 | 45.83 | 130 |
|  |  |  | Reverse | GTCAATTGTCTGAATTTCACATCTGG |  | 59.19 | 38.46 |  |
|  | *TNFa* | Tumor necrosis factor alpha | Forward | ACCACGCTCTTCTGCCTACT | NM_214022.1 | 60.90 | 55.00 | 132 |
|  |  |  | Reverse | GACGGGCTTATCTGAGGTTTGA |  | 60.09 | 50.00 |  |
|  | *IFNg* | Interferon gamma | Forward | AAGAATTGGAAAGAGGAGAGTGACA | NM_213948.1 | 59.93 | 40.00 | 101 |
|  |  |  | Reverse | TGAATGGCCTGGTTATCTTTGA |  | 57.69 | 40.91 |  |
|  | *IFNGR1* | Interferon gamma receptor 1 | Forward | CATGTTACCCAAATCTTTGCTGTCT | NM_001177907.1 | 60.05 | 40.00 | 149 |
|  |  |  | Reverse | CAGTATGCACGCTTGAAATTGTC |  | 59.15 | 43.48 |  |
|  | *HSPA4* | Heat shock protein 70 | Forward | TCTCAATTGCCTGCGATTAATGAA | XM_005661654.3 | 59.36 | 37.50 | 127 |
|  |  |  | Reverse | AGAATGCCCCATGTCTACAAAAAC |  | 59.78 | 41.67 |  |
|  | *CCL20* | Chemokine (C-C motif) ligand 20 | Forward | AGACCATATTCTTCACCCCAGATTT | NM_001024589.1 | 59.81 | 40.00 | 113 |
|  |  |  | Reverse | CACACACGGCTAACTTTTTCTTTG |  | 59.50 | 41.67 |  |
|  | *REG3G* | Regenerating-islet derived protein 3 gamma | Forward | TGCCTGATGCTCCTGTCTCA | NM_001144847.1 | 60.91 | 55.00 | 111 |
|  |  |  | Reverse | GGCATAGCAGTAGGAAGCATAGG |  | 60.62 | 52.17 |  |
|  | *FAXDC2* | Fatty acid hydrolase domain containing 2 | Forward | CCATGACTACCACCATCTCAAGTT | XM_005672602.2 | 60.32 | 45.83 | 121 |
|  |  |  | Reverse | CAGGATCGTGTGTCTCTCGTA |  | 58.99 | 52.38 |  |
|  | *GBP1* | Guanylate binding protein 1 | Forward | GAATCCATCACAGCAGACGAGTA | NM_001128473.1 | 60.18 | 47.83 | 99 |
|  |  |  | Reverse | GATACAGAGTCGAGGCAGGTTAA |  | 59.62 | 47.83 |  |
|  | *DEFB1* | Porcine beta-defensin 1 | Forward | GTATTCCTCCTCATGGTCCTGTT | NM_213838.1 | 59.54 | 47.83 | 134 |
|  |  |  | Reverse | CAGGTGCCGATCTGTTTCATC |  | 59.33 | 52.38 |  |
|  | *pBD2* | Porcine beta-defensin 2 | Forward | ACTGTCTGCCTCCTCTCTTCC | NM_214442.2 | 60.89 | 57.14 | 154 |
|  |  |  | Reverse | TGTAACAGGTCCCTTCAATCCTG |  | 59.99 | 47.83 |  |
|  | *pBD3* | Porcine beta-defensin 3 | Forward | ACCTTCTCTTTGCCTTGCTCTT | NM_214444.1 | 60.16 | 45.45 | 164 |
|  |  |  | Reverse | GCCACTCACAGAACAGCTACC |  | 60.94 | 57.14 |  |
|  | *IKBKB* | Inhibitor of nuclear factor kappa B kinase subunit beta | Forward | TGGGATCACATCGGACAAACTG | NM_001099935.1 | 60.62 | 50.00 | 85 |
|  |  |  | Reverse | CTTCACCTCGTTCTCCCGTC |  | 60.11 | 60.00 |  |
|  | *NFKB1* | Nuclear factor kappa B subunit 1 | Forward | TCCACAAGGCAGCAAATAGA | NM_001048232.1 | 57.12 | 45.00 | 83 |
|  |  |  | Reverse | AAGCTGAGTTTGCGAAAGGA |  | 58.03 | 45.00 |  |
|  | *NFKBIA* | NFKB inhibitor alpha | Forward | GAGGATGAGCTGCCCTATGAC | NM_001005150.1 | 60.00 | 57.14 | 85 |
|  |  |  | Reverse | CCATGGTCTTTTAGACACTTTCC |  | 57.37 | 43.48 |  |
|  | *TGFB1* | Transforming growth factor beta 1 | Forward | CTGGCCCCCAGTGACTCA | NM_214015.2 | 60.93 | 66.67 | 96 |
|  |  |  | Reverse | GCGAAAACCCTCTATAGCCTCTCT |  | 61.77 | 50.00 |  |
| Nutrient transporters | *SLC5A1* | Solute carrier family 5 (sodium/glucose cotransporter) member 1 | Forward | GGCCATCTTTCTCTTACTGGCA | NM_001164021.1 | 60.36 | 50.00 | 147 |
|  |  |  | Reverse | CCTCCCACTTCATGAAAAGCAAAC |  | 60.80 | 45.83 |  |
|  | *SLC7A8* | Solute carrier family 7 (amino acid transporter light chain, L System) member 8 | Forward | GTCGCTTATGTCACTGCAATGT | XM_021099239.1 | 59.58 | 45.45 | 122 |
|  |  |  | Reverse | GACAGGGCGACGGAAATG |  | 58.51 | 61.11 |  |
|  | *SLC16A1* | Monocarboxylate transporter 1 | Forward | CCTTGTTGGACCTCAGAGATTCTC | NM_001128445.1 | 60.62 | 50.00 | 132 |
|  |  |  | Reverse | CAGTATGTGTATTTATAGTCTCCGTATATGTC |  | 59.69 | 34.38 |  |
|  | *SLC39A4* | Solute carrier family 39 (zinc transporter) member 4 | Forward | ATCTTTGGGCTCTTGCTCCTT | XM_001925360.5 | 59.64 | 47.62 | 139 |
|  |  |  | Reverse | GCAGCCCCAGCACCTTAG |  | 60.44 | 66.67 |  |
| Stress | *HSD11B1* | Hydroxysteroid (11-beta) dehydrogenase 1 | Forward | GTCAGAAGAAACTCTCAAGAAGGTG | NM_214248.3 | 59.53 | 44.00 | 99 |
|  |  |  | Reverse | GCGAAGGTCATGTCCTCCAT |  | 59.82 | 55.00 |  |
| Reference genes | *GAPDH* | Glyceraldehyde-3-phosphate dehydrogenase | Forward | TTCGTCAAGCTCATTTCCTGGTA | NM_001206359.1 | 59.99 | 43.48 | 129 |
|  |  |  | Reverse | CCTCGCGTGCTCTTGCT |  | 60.09 | 64.71 |  |
|  | *ACTB* | Beta-actin | Forward | AAGGACCTCTACGCCAACAC | XM_021086047.1 | 59.68 | 55.00 | 130 |
|  |  |  | Reverse | CTGGAGGCGCGATGATCTT |  | 59.93 | 57.89 |  |
|  | *TBP* | TATA-box binding protein | Forward | ACAGAATGATCAAACCGAGAATTGT | XM_021085497.1 | 59.29 | 36.00 | 80 |
|  |  |  | Reverse | TGCTCTGACTTTAGCACCTGTTAA |  | 60.20 | 41.67 |  |
|  | *HPRT1* | Hypoxanthine phosphoribosyltransferase 1 | Forward | TCATTATGCCGAGGATTTGGA | NM_001032376.2 | 57.14 | 42.86 | 91 |
|  |  |  | Reverse | CTCTTTCATCACATCTCGAGCAA |  | 58.82 | 43.48 |  |

TM – Melting Temperature, GC% - percentage of guanine and cytosine nucleotides in the primer sequence
